# A Labeled GM-PHD Filter for Explicitly Tracking Multiple Targets †

**DOI:** 10.3390/s21113932

**Published:** 2021-06-07

**Authors:** Yiyue Gao, Defu Jiang, Chao Zhang, Su Guo

**Affiliations:** 1 College of Energy and Electrical Engineering, Hohai University, Nanjing 210098, China; gyiyue@hhu.edu.cn (Y.G.); 20060067@hhu.edu.cn (S.G.); 2 Laboratory of Array and Information Processing, College of Computer and Information, Hohai University, Nanjing 210098, China; zhangchao2002@hhu.edu.cn

**Keywords:** multitarget tracking, probability hypothesis density filter, Gaussian mixture, track continuity, state extraction

## Abstract

In this study, an explicit track continuity algorithm is proposed for multitarget tracking (MTT) based on the Gaussian mixture (GM) implementation of the probability hypothesis density (PHD) filter. Trajectory maintenance and multitarget state extraction in the GM-PHD filter have not been effectively integrated to date. To address this problem, we propose an improved GM-PHD filter. In this approach, the Gaussian components are classified and labeled, and multitarget state extraction is converted into multiple single-state extractions. This provides the identity label of the individual target and can shield against the negative effects of clutter in the prior density region on the estimates, thus realizing the integration of trajectory maintenance with state extraction in the GM-PHD filter. As no additional associated procedures are required, the overall real-time performance of the proposed filter is similar to or slightly lower than that of the basic GM-PHD filter. The results of numerical experiments demonstrate that the proposed approach can achieve explicit track continuity.

## 1. Introduction

### 1.1. Background and Motivation

Multitarget tracking (MTT) jointly estimates the target state and the number of targets simultaneously from a sequence of measurements [1]. In MTT, the time-varying number of targets causes data associations between state and measurement sets of targets, which are uncertain. The key challenges encountered involve detection uncertainty, clutter, and data association uncertainty. Therefore, accurately extracting the states of targets and their trajectories has become a critical issue and a hot topic in the field of MTT. The most popular technologies for MTT are the joint probabilistic data association (JPDA) [2,3], multiple hypothesis tracking (MHT) [4], and random finite set (RFS) [1,5].

Since RFS avoids the traditional data association, it has attracted significant attention. Based on RFS and under the framework of Bayesian filtering, many approximations of Bayes multitarget filters have been proposed, mainly including a probability hypothesis density (PHD) filter [1,6,7], cardinalized PHD (CPHD) filter [8], and multi-Bernoulli (MeMBer) filter [9]. These filters are not multitarget trackers, which only estimate target states at individual time instants as opposed to multitarget trajectories. While these filters were not designed with the aim of estimating the trajectories of targets [10], they have been used in many applications [11,12,13].

To provide the information of each track, the notion of labeled RFS is introduced in [14,15]. The generalized labeled multi-Bernoulli (GLMB) filter [14,15] leads to an analytic solution to the Bayes multitarget tracker, which significantly improves the accuracy of multitarget state extraction and explicitly accommodates the estimation of target trajectories. Recently, labeled RFS-based filters and smoothers [10,16,17,18,19] have been further developed, for generating track estimates, which is also the focus of this paper.

A Gibbs-GLMB filter [10] is a computational efficient implementation of the GLMB filter that combines the prediction and update into a single step. A forward-backward labeled multi-Bernoulli smoother in [17] is proposed for MTT, which can improve both the cardinality estimation and the state estimation, and the major computational complexity is linear with the number of targets. The trajectory Poisson multi-Bernoulli filter and the trajectory Poisson multi-Bernoulli mixture filter [18] have better filtering accuracy and real-time performance than those of the Gibbs-GLMB filter. A onetime step lagged Bayes multitarget smoother using the δ-GLMB density [19], which also inherently outputs targets trajectories, outperforms the Gibbs-GLMB filter on both the estimates and target number and state at the cost of higher computational complexity. However, when targets are in a dense clutter, or misdetected, generating the correct multitarget trajectories is difficult in [16,17,18,19], although the computational complexity problem has been improved in [17,18].

Thus, it is necessary to design a relatively low computing cost filter that can provide explicit trajectories whilst considering the intense clutter environment. 

### 1.2. Brief Survey of Related Work

From the viewpoint of practical applicability, the PHD filter is suitable for applications demanding real-time results [20,21,22]. Unlike the full Bayesian filter, the PHD filter iteratively propagates the first moment of the multitarget posterior density. It is a promising technique in terms of computational complexity to solve the MTT problem. However, it does not explicitly accommodate the trajectories of targets [10,21,23]. Furthermore, the PHD filter is known to produce highly uncertain estimates of the target number [21,23]. In recent years, several modifications have been proposed for the trajectories of targets [22,24,25,26,27,28,29,30].

In [29], an explicit track continuity algorithm for the sequence Monte Carlo PHD (SMC-PHD) filter [31] is proposed, which not only shields against the negative effects of clutter but provides the identities of the individual targets. However, when several newborn targets appear simultaneously in one newborn region, it is unable to extract the state estimates of different observations from these newborn targets. Compared to the SMC-PHD filter, the Gaussian mixture PHD (GM-PHD) filter [32] has the advantages of simple state extraction and low computational cost, which is more suitable for the requirement of real-time scenes. Thus, the focus of this study is the GM-PHD filter.

For the estimation of the target trajectory of the GM-PHD filter, recently proposed solutions involve integrating the track identity into the GM-PHD filter. In each iteration process, each Gaussian component is assigned a label to indicate its correlation with one track. These methods can be mainly classified into two groups. One group [24,25] involves running an additional association method based on the estimates outputted by the filter. The other solution [26,27,28,29] extends a label to the state estimate. However, when targets are in close proximity to each other, in a dense clutter, or misdetected, maintaining the correct multitarget trajectories is difficult, although the accuracy of the estimates of the target number has been increased significantly by improving multitarget state extraction strategies [33,34,35,36,37]. Thus, one can conclude that in the GM-PHD filter, track maintenance and multitarget state extraction have not been effectively integrated. Recently, a Gaussian mixture trajectory PHD filter was proposed in [30], which is able to provide trajectory estimates for live targets, without adding labels or tags. It adapts the estimator for the GMPHD filter for the sets of trajectories; thus, its tracking accuracy is the same as that of the PHD filter. When clutters appear in the high prior density region of the target, it is difficult to shield against the interference of clutters.

An instructive idea is that the Gaussian components approximating the posterior information of one target have the same identity label, which will not be changed throughout the overall filtering process. In the multitarget state extraction, only one state estimate can be extracted from the updated survival Gaussian components with the same label. Then, the explicit trajectories can be obtained by simply connecting the state estimates with the same label, which requires no additional track management. To achieve this concept, in this paper, we propose an explicit track continuity algorithm for the GM-PHD filter. 

### 1.3. Main Contributions

The work in this paper is based on a published conference paper [38]. The major contributions of this paper are as follows:

- First, each Gaussian component is assigned a label, and the identity labels of Gaussian components are divided into three classes; once it is determined as a confirmed component, its label will be unchanged throughout the whole filtering process. This is convenient for track maintenance; that is, state estimates with the same label at different times belong to the same track.
- Second, based on the measurements selected by eliminating most clutter, the updated components are obtained, and their parameters are stored in four different query tables. Based only on these query tables, the multitarget state extraction is converted to multiple single-target state extractions providing the identity label, and the negative effects of clutter in the prior density region on the estimates can be prevented. Thus, we realize the integration of trajectory maintenance with state extraction in the GM-PHD filter.
- Third, since the component updating process is based on the selected measurements and no additional associated process is required, the overall real-time performance of the proposed filter is similar to or slightly lower than that of the basic GM-PHD filter. The performance of the proposed approach is verified by simulations designed in a linear scenario.

The remainder of this paper is organized as follows. The technical background is provided in Section 2, while the specific designing of the improved GM-PHD filter is presented in Section 3. The quantitative experiments are described in Section 4, and concluding remarks are provided in Section 5.

## 2. Background

### 2.1. GM-PHD Filter

In MTT, the state and observation sets are time-varying and unknown. The collection of the states of targets at time k is represented as an RFS $X_{k}=\left\{x_{k}^{\left(1\right)},\ldots ,x_{k}^{\left(i\right)},\ldots ,x_{k}^{\left(|X_{k}|\right)}\right\}$, where $\left|X_{k}\right|$ denotes the number of targets, and $x_{k}^{\left(i\right)}$ denotes the state of an individual target. Similarly, the collection of available measurements is an RFS $Z_{k}=\left\{z_{k}^{\left(1\right)},\ldots ,z_{k}^{\left(i\right)},\ldots ,z_{k}^{\left(\left|Z_{k}\right|\right)}\right\}$, where $\left|Z_{k}\right|$ is the number of measurements and $z_{k}^{\left(i\right)}$ is a measurement from a target or due to a clutter. The PHD [6] is the first-order moment of multitarget posterior density.

Assuming that each target follows a linear Gaussian dynamical model and the sensor has a linear Gaussian measurement model [32],

(1) $$f_{k|k-1}(x_{k}|x_{k-1})=N(x_{k};F_{k-1}x_{k-1},Q_{k-1})$$

(2) $$g_{k}(z_{k}|x_{k})=N(z_{k};H_{k}x_{k},R_{k})$$

where N(⋅;m,P) denotes the Gaussian density with mean m and covariance P, $F_{k-1}$ is the state transition matrix, $Q_{k-1}$ is the process noise covariance, $H_{k}$ is the observation matrix, and $R_{k}$ is the measurement noise covariance.

The birth intensity function of the new targets at time k [32] is $\gamma_{k}(x)=\sum_{i=1}^{J_{\gamma ,k}}w_{\gamma ,k}^{\left(i\right)}N\left(x;m_{\gamma ,k}^{\left(i\right)},P_{\gamma ,k}^{\left(i\right)}\right)$, where $J_{\gamma ,k}$ denotes the number of newborn Gaussian components, and $w_{\gamma ,k}^{\left(i\right)}$, $m_{\gamma ,k}^{\left(i\right)}$, and $P_{\gamma ,k}^{\left(i\right)}$ are the weight, mean, and covariance, respectively, of the ith newborn Gaussian component. Suppose that the survival probability $p_{S,k}=p_{S,k}(x_{k})$, the detection probability $p_{D,k}=p_{D,k}(x_{k})$. Based on these assumptions [32], the PHD filter can be efficiently approximated by mixed Gaussian components. w is the weight of Gaussian density and the posterior intensity $D_{k-1|k-1}(x)$ at time k−1 [32] is a Gaussian mixture form with $J_{k-1}$ survival components as follows:

(3) $$D_{k-1|k-1}(x)=\sum_{i=1}^{J_{k-1}}w_{k-1}^{\left(i\right)}N\left(x;m_{k-1}^{\left(i\right)},P_{k-1}^{\left(i\right)}\right)$$

The predicted intensity $D_{k|k-1}(x)$ at time k is also a Gaussian mixture with $J_{k|k-1}$ components calculated as follows:

(4) $$\begin{array}{cc} D_{k|k-1}(x) & =\sum_{i=1}^{J_{k|k-1}}w_{k|k-1}^{\left(i\right)}N\left(x;m_{k|k-1}^{\left(i\right)},P_{k|k-1}^{\left(i\right)}\right)\\ & =D_{S,k|k-1}(x)+\gamma_{k}(x) \end{array}$$

where $D_{S,k|k-1}(x)=\sum_{i=1}^{J_{k-1}}w_{S,k|k-1}^{\left(i\right)}N\left(x;m_{S,k|k-1}^{\left(i\right)},P_{S,k|k-1}^{\left(i\right)}\right)$ is the intensity of survival targets, $w_{S,k|k-1}^{\left(i\right)}=p_{S,k}w_{k-1}^{\left(i\right)}$, $m_{S,k|k-1}^{\left(i\right)}=F_{k-1}m_{k-1}^{\left(i\right)}$, $P_{S,k|k-1}^{\left(i\right)}=F_{k-1}P_{k-1}^{\left(i\right)}{\left(F_{k-1}\right)}^{T}+Q_{k-1}$.

When a new set $Z_{k}$ arrives, the posterior intensity $D_{k|k}$ at time k [32] is updated and can be described as follows:

(5) $$D_{k|k}(x)=(1-p_{D,k})D_{k|k-1}(x)+\sum_{z\in Z_{k}}D_{D,k}(x;z)$$

where

(6) $$D_{D,k}(x;z)=\sum_{i=1}^{J_{k|k-1}}w_{k|k}^{\left(i\right)}(z)N\left(x;m_{k|k}^{\left(i\right)}(z),P_{k|k}^{\left(i\right)}\right)$$

(7) $$w_{k|k}^{\left(i\right)}(z)=\frac{p_{D,k}w_{k|k-1}^{\left(i\right)}g_{k}(z|m_{k|k-1}^{\left(i\right)})}{\lambda c(z)+p_{D,k}\sum_{l=1}^{J_{k|k-1}}w_{k|k-1}^{\left(l\right)}g_{k}(z|m_{k|k-1}^{\left(l\right)})}$$

(8) $$g_{k}(z|m_{k|k-1}^{\left(i\right)})=N(z;H_{k}m_{k|k-1}^{\left(i\right)},S_{k}^{\left(i\right)})$$

(9) $$m_{k|k}^{\left(i\right)}(z)=m_{k|k-1}^{\left(i\right)}+K_{k}^{\left(i\right)}\left(z-H_{k}m_{k|k-1}^{\left(i\right)}\right)$$

(10) $$P_{k|k}^{\left(i\right)}=\left[I-K_{k}^{\left(i\right)}H_{k}\right]P_{k|k-1}^{\left(i\right)}$$

(11) $$S_{k}^{\left(i\right)}=R_{k}+H_{k}P_{k|k-1}^{\left(i\right)}{\left(H_{k}\right)}^{T}$$

(12) $$K_{k}^{\left(i\right)}=P_{k|k-1}^{\left(i\right)}{\left(H_{k}\right)}^{T}{\left(H_{k}P_{k|k-1}^{\left(i\right)}{\left(H_{k}\right)}^{T}+R_{k}\right)}^{-1}$$

For more details on the GM-PHD filter, we refer the reader to the original study [32].

To reduce the computational load, the increasing Gaussian components must be pruned and merged. Then, the posterior intensity $D_{k|k}$ is represented as follows:

(13) $$D_{k|k}(x)=\sum_{i=1}^{J_{k}}w_{k}^{\left(i\right)}N\left(x;m_{k}^{\left(i\right)},P_{k}^{\left(i\right)}\right)$$

**Figure 1:**
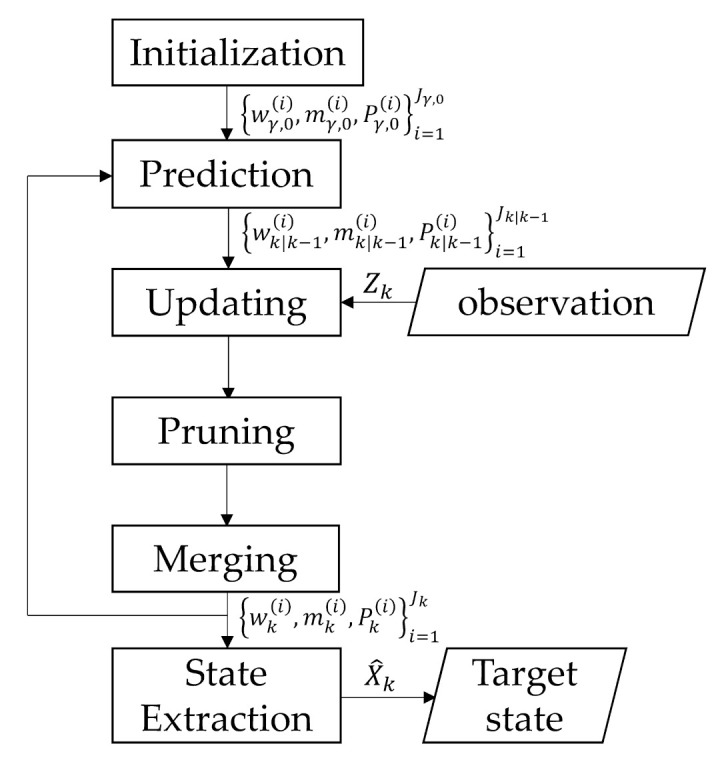
Overall procedure of the basic GM-PHD filter.

The multiple-target state estimates can be extracted based on the threshold $w_{th}$ (this is generally 0.5) from $D_{k|k}(x)$. Figure 1 shows the overall procedure of the basic GM-PHD filter.

### 2.2. Problem Formulation

**Figure 2:**
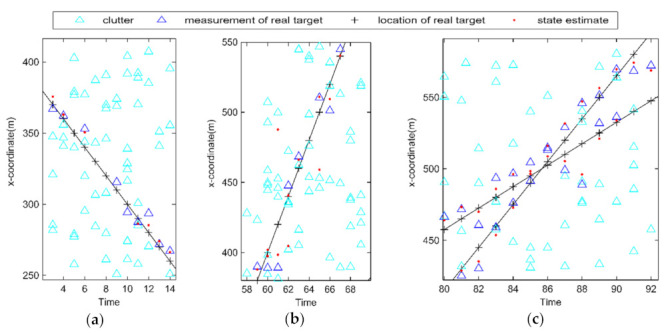
Phenomena affecting track continuity of the basic GM-PHD filter. (**a**) detection uncertainty; (**b**) false state estimates; (**c**) closely spaced targets.

The basic GM-PHD filter in Figure 1 does not explicitly accommodate target trajectory estimation. Trajectory continuity and state estimates essentially influence and interact with each other. In MTT, detection uncertainty, false state estimates, and closely spaced targets have detrimental effects on track continuity. Figure 2 graphically depicts these phenomena.

As shown in Figure 2, the clutter rate was relatively high, and the detection probability was 0.9 in the current tracking scenario. In Figure 2a, at times *k* = 7 and *k* = 8, the target was misdetected, and its posterior intensity was weakened. This caused its inability to obtain timely state estimates even if the target was redetected at times *k* = 9 and *k* = 10. Managing the trajectories of the state estimates at times *k* = 6 and *k* = 11 was a challenge. In Figure 2b, at times *k* = 60, *k* = 61, *k* = 62, and *k* = 65, there were false state estimates caused by clutter points appearing in the high-prior density region of the target. In Figure 2c, two targets were closely spaced, and their state estimates were nearby. It is difficult to identify the state estimate with which they should be associated. The above phenomena make it difficult to provide a correct target trajectory based on the basic GM-PHD filter.

A trajectory is a time-sequence of state estimates with the same label, such as $\left\{\left({\hat{x}}^{}{}_{k-1},\ell \right),\left({\hat{x}}^{}{}_{k},\ell \right),\left({\hat{x}}^{}{}_{k+2},\ell \right)\right\}$, wherein ℓ provides the means to identify the trajectory or track of one target. Furthermore, even if there is no state estimate at time *k* + 1, the correct track continuity can still be obtained. To achieve explicit trajectories based on the GM-PHD filter, we proposed an algorithm that labels all Gaussian components, which we called the labeled GM-PHD (LGM-PHD) filter.

## 3. The Proposed Multitarget Tracking Algorithm

Assuming the target birth intensity is known a priori, a “one-to-one” principle for the multitarget state extraction of the GM-PHD filter was proposed herein.

The “one-to-one” principle for the GM-PHD filter: In the multitarget state extraction, only one state estimate can be extracted from the updated Gaussian components with the same label, originating from the survival ones.

To abide by the “one-to-one” principle, each Gaussian component is assigned a label, and these labels are divided into three categories in the LGM-PHD filter. In this paper, the identity labels of the Gaussian components are divided into three classes, the labels of newborn Gaussian components, the labels of unconfirmed Gaussian components (approximating the posterior information of targets having their own unconfirmed trajectory), and the labels of confirmed components (approximating the posterior information of targets having their own confirmed trajectory). The attribute of a label ℓ is determined based on the value range as follows:

(14) $$\begin{array}{c} \mathrm{label}\;\mathrm{of}\;a\;\mathrm{newborn}\;\mathrm{component},\;\;\mathrm{if}\;\ell \geq V_{new},\\ \mathrm{label}\;\mathrm{of}\;\mathrm{an}\;\mathrm{unconfirmed}\;\mathrm{component},\;\mathrm{if}\;V_{un}\leq \ell <V_{new},\\ \mathrm{label}\;\mathrm{of}\;a\;\mathrm{confirmed}\;\mathrm{component},\;\mathrm{if}\;0\leq \ell <V_{un}. \end{array}$$

where $V_{un}$ is a value far greater than the total number of targets that could possibly appear in this scenario, $V_{new}\neq V_{un}$, and $V_{new}\gg V_{un}$. In general, $V_{un}=200$ and $V_{new}=400$.

$r_{\max}$ and $r_{\max t}$ are used to count the confirmed and unconfirmed trajectories, respectively. At time *k* = 0, $r_{\max}$ is initialized as 0 and $r_{\max t}$ is initialized as $V_{un}$. When a new confirmed trajectory is initiated, $r_{\max}$ is incremented by one; when a new unconfirmed trajectory is initiated, $r_{\max t}$ is also incremented by one.

**Figure 3:**
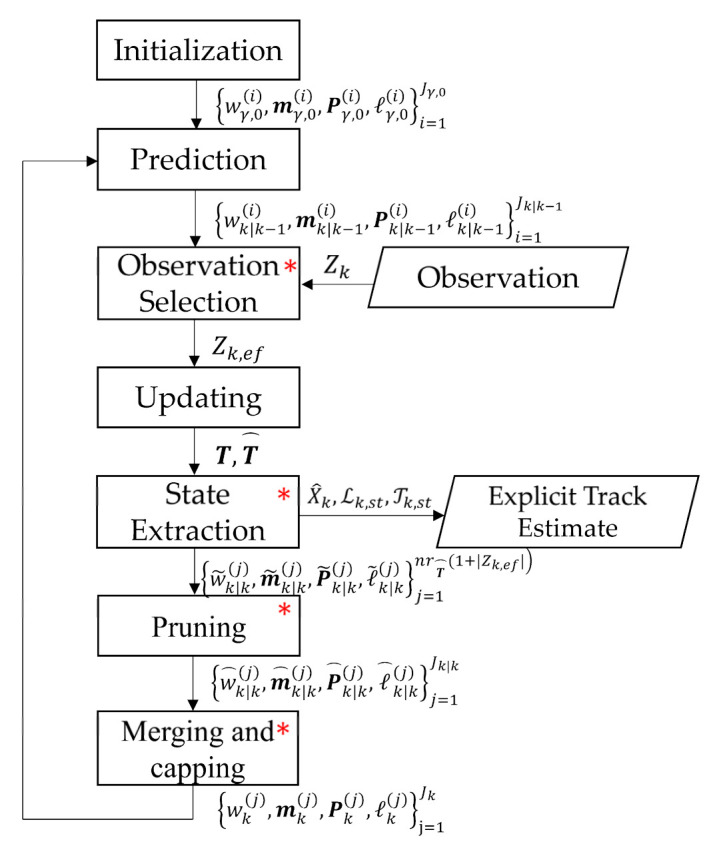
Overall procedure of the LGM-PHD filter.

The overall procedure of the LGM-PHD filter contains seven steps—initialization, prediction, observation selection, updating, state extraction, pruning, and merging and capping—as shown in Figure 3. When compared with the basic GM-PHD filter in Figure 1, the submodules marked with “∗” are either modified or proposed in this paper.

### 3.1. Initialization and Prediction

At time k, newborn targets appear spontaneously according to a Poisson point process with the intensity function

(15) $$\gamma_{k}(x)=\sum_{i=1}^{J_{\gamma ,k}}w_{\gamma ,k}^{\left(i\right)}N\left(x;m_{\gamma ,k}^{\left(i\right)},P_{\gamma ,k}^{\left(i\right)}\right)$$

where each newborn Gaussian component is assigned a label $\ell_{\gamma ,k}^{\left(i\right)}=V_{new}+i$, $i=1,\ldots ,J_{\gamma ,k}$. According to (15), the initial intensity $D_{0|0}$ is described as follows: ${\left\{w_{\gamma ,0}^{\left(i\right)},m_{\gamma ,0}^{\left(i\right)},P_{\gamma ,0}^{\left(i\right)},\ell_{\gamma ,0}^{\left(i\right)}\right\}}_{i=1}^{J_{\gamma ,0}}$.

With this assumption, at time *k* − 1, the posterior probability density $D_{k-1|k-1}(x)$ is approximated by ${\left\{w_{k-1}^{\left(j\right)},m_{k-1}^{\left(j\right)},P_{k-1}^{\left(j\right)},\ell_{k-1}^{\left(j\right)}\right\}}_{j=1}^{J_{k-1}}$, where $\ell_{k-1}^{\left(j\right)}$ is the identity label of the jth survival Gaussian component. Then, according to (4), the priori density at time k is approximated by ${\left\{w_{k|k-1}^{\left(i\right)},m_{k|k-1}^{\left(i\right)},P_{k|k-1}^{\left(i\right)},\ell_{k|k-1}^{\left(i\right)}\right\}}_{i=1}^{J_{k|k-1}}$, where $J_{k|k-1}=J_{k-1}+J_{\gamma ,k}$. The ith predicted Gaussian component is labeled as follows:

(16) $$\ell_{k|k-1}^{\left(i\right)}=\left\{ \begin{array}{l} \ell_{k-1}^{\left(i\right)},\begin{array}{cc} & i=1,\ldots ,J_{k-1} \end{array}\\ \ell_{\gamma ,k}^{\left(i-J_{k-1}\right)},\begin{array}{cc} & i=J_{k-1}+1,\ldots ,J_{k|k-1} \end{array} \end{array}\right.$$

To obtain the time-sequence of states with the same label, the labels of conformed components remain unchanged throughout the overall filtering process. In addition, the labels of all types of components can be inherited, and only components with the same label can be merged.

### 3.2. Observation Selection

To achieve robust track maintenance, clutter should be eliminated. Considering the overall real-time performance of the filter, it is necessary to eliminate most clutter before the updating procedure, to select effective observations. Given the zero-mean-measurement Gaussian white noise with the covariance matrix $R_{k}=diag({\left[\begin{array}{cc} \sigma_{x}^{2} & \sigma_{y}^{2} \end{array}\right]}^{T})$, the threshold d(a) is calculated by $d\left(a\right)=a\left\|{\left[\begin{array}{cc} \sigma_{x}^{} & \sigma_{y}^{} \end{array}\right]}^{T}-{\left[\begin{array}{cc} 0 & 0 \end{array}\right]}^{T}\right\|$, in which ‖⋅‖ is the Euclidean distance and a is the confidence coefficient. In this gating algorithm, a=6. Initializing the effective observation set $Z_{k,ef}$ as ∅, based on all the predicted Gaussian components at time k, $Z_{k,ef}={\left\{z_{k,ef}^{\left(j\right)}\right\}}_{j=1}^{\left|Z_{k,ef}\right|}$ is obtained by the operations in Algorithm 1.
**Algorithm 1.** The observation selection for the GM-PHD filter algorithm1: **Given**
${\left\{w_{k|k-1}^{\left(i\right)},m_{k|k-1}^{\left(i\right)},P_{k|k-1}^{\left(i\right)}\right\}}_{i=1}^{J_{k|k-1}}$
**and**
$Z_{k}={\left\{z_{k}^{\left(j\right)}\right\}}_{j=1}^{\left|Z_{k}\right|}$
2: **Initialization**
$Z_{k,ef}=\emptyset$
3: **for**
$j=1,\ldots ,\left|Z_{k}\right|$
**do**
4:  **for**
$i=1,\ldots ,J_{k|k-1}$
**do**
5:     **–** $d^{\left(j,i\right)}=\left\|z_{k}^{\left(j\right)}-H_{k}m_{k|k-1}^{\left(i\right)}\right\|$
6:     **–**
**if**
$d^{\left(j,i\right)}\leq d\left(a\right)$
7:     **–** $Z_{k,ef}=\left[Z_{k,ef},z_{k}^{\left(j\right)}\right]$
8:     **–**
**break**9:    **–**
**end**10:   **end**11:  **end**

### 3.3. Updating

First, four tables $U_{w}$, $U_{m}$, $U_{P}$, and $U_{\ell }$, all with size $J_{k|k-1}\times (1+\left|Z_{k,ef}\right|)$, are created to store parameters w, m, P, and ℓ of each updated Gaussian component. They are uniformly called query table U. 

Second, based on $Z_{k,ef}$, the posterior intensity $D_{k|k}$ at time k can be described as follows:$D_{k|k}(x)=(1-p_{D,k})D_{k|k-1}(x)+\sum_{z_{k,ef}^{\left(j\right)}\in Z_{k,ef}}D_{D,k}(x;z_{k,ef}^{\left(j\right)})$, where $D_{D,k}(x;z_{k,ef}^{\left(j\right)})=\sum_{i=1}^{J_{k|k-1}}w_{k|k}^{\left(i\right)}(z_{k,ef}^{\left(j\right)})N\left(x;m_{k|k}^{\left(i\right)}(z_{k,ef}^{\left(j\right)}),P_{k|k}^{\left(i\right)}\right)$. Based on the ith predicted Gaussian component and $z_{k,ef}^{\left(j\right)}$, we obtain the parameter set $\left\{w_{k|k}^{\left(i\right)}(z_{k,ef}^{\left(j\right)}),m_{k|k}^{\left(i\right)}(z_{k,ef}^{\left(j\right)}),P_{k|k}^{\left(i\right)},\ell_{k|k}^{\left(i\right)}\right\}$ of the updated Gaussian component via (5)–(12), where $\ell_{k|k}^{\left(i\right)}=\ell_{k|k-1}^{\left(i\right)}$. Based on this parameter set, the elements in the ith row and the (j+1)th column of $U_{w}$, $U_{m}$, $U_{P}$, and $U_{\ell }$ are obtained as follows:

(17) $$\begin{array}{c} U_{w}(i,j+1)=w_{k|k}^{\left(i\right)}(z_{k,ef}^{\left(j\right)})\\ U_{m}(i,j+1)=m_{k|k}^{\left(i\right)}(z_{k,ef}^{\left(j\right)})\\ U_{P}(i,j+1)=P_{k|k}^{\left(i\right)}\\ U_{\ell }(i,j+1)=\ell_{k|k}^{\left(i\right)} \end{array}$$

$\left(1-p_{D,k})D_{k|k-1}(x\right)$ is approximated by the parameter set ${\left\{(1-p_{D,k})w_{k|k-1}^{\left(i\right)},m_{k|k-1}^{\left(i\right)},P_{k|k-1}^{\left(i\right)},\ell_{k|k-1}^{\left(i\right)}\right\}}_{i=1}^{J_{k|k-1}}$. Based on this parameter set, the elements in the ith row and the 1th column of $U_{w}$, $U_{m}$, $U_{P}$, and $U_{\ell }$ are obtained as follows:

(18) $$\begin{array}{c} U_{w}(i,1)=(1-p_{D,k})w_{k|k-1}^{\left(i\right)}\\ U_{m}(i,1)=m_{k|k-1}^{\left(i\right)}\\ U_{P}(i,1)=P_{k|k-1}^{\left(i\right)}\\ U_{\ell }(i,1)=\ell_{k|k-1}^{\left(i\right)} \end{array}$$

Third, four tables, ${\overset{⌢}{U}}_{w}$, ${\overset{⌢}{U}}_{m}$, ${\overset{⌢}{U}}_{P}$, and ${\overset{⌢}{U}}_{\ell }$, are created, which are collectively called the $\overset{⌢}{U}$

(19) $${\overset{⌢}{U}}_{w}=U_{w},{\overset{⌢}{U}}_{m}=U_{m},{\overset{⌢}{U}}_{P}=U_{P},{\overset{⌢}{U}}_{\ell }=U_{\ell }$$

When a new confirmed trajectory or a new unconfirmed trajectory is established, its information is documented and stored in $\overset{⌢}{U}$.

### 3.4. State Extraction

In this section, by means of U, certain Gaussian components with the same label are associated with one effective observation, and if the maximum weight in these components satisfies some conditions, one state estimate is extracted.

Complete target status information contains state estimate $\hat{x}$, track label ℓ, and occurrence time k. Thus, at time k, the state estimate set ${\hat{X}}_{k}$, the track label set ${ℒ}_{k,st}$, and the occurrence time set $T_{k,st}$ are all initialized as ∅. The specific steps in state extraction process are as follows.

Step 1: First, obtain the maximum value $\bar{w}$ in $U_{w}(:,2:end)$, where $U_{w}(:,2:end)$ represents the elements from the second column to the last column of each row in $U_{w}$. $U_{w}(i,j)$ denotes the element in the jth column of the ith row in $U_{w}$. Here, $\bar{w}=U_{w}(i^{*},j^{*})$ is the maximum, $\left(i^{*},j^{*})=\arg\max(U_{w}(i,j)\right)$, $\forall i=1,\ldots ,J_{k|k-1}$, and $\forall j=2,\ldots ,1+\left|Z_{k,ef}\right|$. Very rarely, $\bar{w}$ may be in two or more different locations in $U_{w}$.

Next, if $\bar{w}<w_{b}$, jump to Step 4. $w_{b}$ is the threshold of the basic weight. In general, $w_{b}$ is set to 0.02. If $\bar{w}\geq w_{b}$, based on the row and column numbers of $\bar{w}$, obtain the unique label set ${ℒ}_{\bar{w}}={\left\{\ell_{\bar{w}}^{\left(i\right)}=U_{\ell }\left(rn^{\left(i\right)},cn^{\left(i\right)}\right)\right\}}_{i=1}^{\left|{ℒ}_{\bar{w}}\right|}$, and initialize CN=∅. CN is used to store the column numbers of observations based on which some tracks will be established or maintained. In most cases, the label set only has one element, i.e., $\left|{ℒ}_{\bar{w}}\right|=1$.

Step 2: For $i=1,\ldots ,\left|{ℒ}_{\bar{w}}\right|$, determine the attribute of $\ell_{\bar{w}}^{\left(i\right)}$, and execute the following actions for the Gaussian components with $\ell_{\bar{w}}^{\left(i\right)}$.

- First, obtain $\hat{x}$ by $\hat{x}=H_{k}U_{m}(rn^{\left(i\right)},cn^{\left(i\right)})$. The row numbers of elements with $\ell_{\bar{w}}^{\left(i\right)}$ in $U_{\ell }$ are denoted as $RN_{\ell_{\bar{w}}^{\left(i\right)}}$.
- Next, the attribute of $\ell_{\bar{w}}^{\left(i\right)}$ will be used to determine which of the following three cases will be executed.

Case A: $\ell_{\bar{w}}^{\left(i\right)}\geq V_{new}$, the label of newborn components.

As several newborn targets may appear simultaneously in one newborn region, newborn Gaussian components with the same label may contain information on several newborn targets. Therefore, several components with large weights in $U_{w}\left(RN_{\ell_{\bar{w}}^{\left(i\right)}},2:(1+\left|Z_{k,ef}\right|)\right)$ can be extracted as the state estimates of different observations, and new tracks can be established accordingly. The specific steps are as follows:

① The sequence number set $CN_{w_{l}}$ of the different observations with large weights in $U_{w}\left(RN_{\ell_{\bar{w}}^{\left(i\right)}},2:(1+\left|Z_{k,ef}\right|)\right)$ is obtained as follows:

(20) $$\begin{array}{c} CN_{w_{l}}^{\prime }=\left\{j|U_{w}\left(RN_{\ell_{\bar{w}}^{\left(i\right)}},j\right)\geq w_{l},\forall j=2,\ldots ,1+\left|Z_{k,ef}\right|\right\}\\ CN_{w_{l}}={\left\{cn_{w_{l}}^{\left(j1\right)}\right\}}_{j1=1}^{\left|CN_{w_{l}}^{}\right|}=uni(CN_{w_{l}}^{\prime }) \end{array}$$

where $w_{l}$ is the threshold of large weights and uni(A) is an operator that obtains the unique elements in set A. In general, $w_{l}$ is set to 0.4.

Additionally, $CN=\left[CN,CN_{w_{l}}\right]$. 

② If $\left|CN_{w_{l}}\right|\geq 1$, for $j1=1,\ldots ,\left|CN_{w_{l}}\right|$, $\left|CN_{w_{l}}\right|$ state estimates will be extracted for the large weight observations and $\left|CN_{w_{l}}\right|$ new confirmed tracks will be established. The j1th target status information is obtained as follows:

(21) $$\begin{array}{c} r_{\max}=r_{\max}+1,\\ rn_{w_{l}}=\arg\underset{i1\in RN_{\ell_{\bar{w}}^{\left(i\right)}}}{\max(}U_{w}\left(i1,cn_{w_{l}}^{\left(j1\right)}\right)),\\ {\hat{X}}_{k}=\left[{\hat{X}}_{k},H_{k}U_{m}\left(rn_{w_{l}},cn_{w_{l}}^{\left(j1\right)}\right)\right],\\ {ℒ}_{k,st}=\left[{ℒ}_{k,st},r_{\max}\right],\\ T_{k,st}=\left[T_{k,st},k\right]. \end{array}$$

In (21), the label of the new confirmed track is given by $r_{\max}=r_{\max}+1$, $rn_{w_{l}}$ is the row number of the maximum weight in $U_{w}\left(RN_{\ell_{\bar{w}}^{\left(i\right)}},cn_{w_{l}}^{\left(j1\right)}\right)$ and the single-target state estimate is $H_{k}U_{m}\left(rn_{w_{l}},cn_{w_{l}}^{\left(j1\right)}\right)$, which is stored in state estimate set ${\hat{X}}_{k}$ and labeled as $r_{\max}$. The information on the j1th new confirmed track is established by modifying ${\overset{⌢}{U}}_{w}$, ${\overset{⌢}{U}}_{m}$, ${\overset{⌢}{U}}_{P}$, and ${\overset{⌢}{U}}_{\ell }$ as follows:

(22) $$\begin{array}{l} \begin{array}{l} {\overset{⌢}{U}}_{w}\left(RN_{\ell_{\bar{w}}^{\left(i\right)}},:\right)=0,\\ {\overset{⌢}{U}}_{w}\left(rn_{w_{l}},cn_{w_{l}}^{\left(j1\right)}\right)=U_{w}\left(rn_{w_{l}},cn_{w_{l}}^{\left(j1\right)}\right),\\ {\overset{⌢}{U}}_{\ell }\left(RN_{\ell_{\bar{w}}^{\left(i\right)}},:\right)=r_{\max}. \end{array}\}\begin{array}{cc} \mathrm{if} & j1=1. \end{array}\\ \begin{array}{l} {\overset{⌢}{U}}_{w}\left(\mathrm{end}+1,:\right)=0,\\ {\overset{⌢}{U}}_{w}\left(\mathrm{end},cn_{w_{l}}^{\left(j1\right)}\right)=U_{w}\left(rn_{w_{l}},cn_{w_{l}}^{\left(j1\right)}\right),\\ {\overset{⌢}{U}}_{\ell }\left(\mathrm{end}+1,:\right)=r_{\max},\\ {\overset{⌢}{U}}_{m}\left(\mathrm{end}+1,:\right)=U_{m}\left(rn_{w_{l}},:\right),\\ {\overset{⌢}{U}}_{P}\left(\mathrm{end}+1,:\right)=U_{P}\left(rn_{w_{l}},:\right). \end{array}\}\begin{array}{cc} \mathrm{if} & j1>1 \end{array}. \end{array}$$

where $\overset{⌢}{U}\left(\mathrm{end},j\right)$ denotes the elements in the jth column of the last row in $\overset{⌢}{U}$, and $\overset{⌢}{U}\left(\mathrm{end}+1,:\right)$ denotes adding one row at the bottom of $\overset{⌢}{U}$. If j1>1, it means at least one state estimate has been extracted at time k. In other words, two or more confirmed trajectories will be simultaneously established from one prior newborn region. Thus, one additional row should be added in $\overset{⌢}{U}$ to establish the posterior information of the individual track. To shield against interference from closely spaced components with another label, only the elements in $U\left(rn_{w_{l}},cn_{w_{l}}^{\left(j1\right)}\right)$ can be reserved.

③ Additionally, some components whose weights are between $w_{s}$ and $w_{l}$ in $U_{w}\left(RN_{\ell_{\bar{w}}^{\left(i\right)}},2:(1+\left|Z_{k,ef}\right|)\right)$ should also be associated with different observations. The sequence number set $CN_{w_{bl}}^{}$ of these observations with small weights is given as follows:

(23) $$\begin{array}{c} {{CN}^{\prime \prime }}_{w_{bl}}=\left\{j|w_{s}\leq U_{w}\left(RN_{\ell_{\bar{w}}^{\left(i\right)}},j\right)<w_{l},\forall j=2,\ldots ,1+\left|Z_{k,ef}\right|\right\}\\ {{CN}^{\prime }}_{w_{bl}}=uni({{CN}^{\prime \prime }}_{w_{bl}})\\ {CN}_{w_{bl}}^{}={\left\{{cn}_{w_{bl}}^{\left(j2\right)}\right\}}_{j2=1}^{\left|CN_{w_{bl}}^{}\right|}={{CN}^{\prime \prime }}_{w_{bl}}-{CN}_{w_{l}}^{} \end{array}$$

where $w_{s}$ is the threshold of small weights. In general, $w_{s}$ is set to 0.1.

Additionally, $CN=\left[CN,CN_{w_{bl}}^{}\right]$.

④ If $\left|CN_{w_{bl}}^{}\right|\geq 1$, for $j2=1,\ldots ,\left|CN_{w_{bl}}^{}\right|$, $\left|CN_{w_{bl}}^{}\right|$ unconfirmed tracks will be established for the small weight observations. The information on the j2th unconfirmed track is obtained by specific steps as follows:

(24) $$r_{\max t}=r_{\max t}+1$$

(25) $$rn_{w_{bl}}=\arg\underset{i1\in RN_{\ell_{\bar{w}}^{\left(i\right)}}}{\max(}U_{w}\left(i1,cn_{w_{bl}}^{\left(j2\right)}\right))\;$$

(26) $$\begin{array}{l} \begin{array}{l} {\overset{⌢}{U}}_{w}\left(RN_{\ell_{\bar{w}}^{\left(i\right)}},:\right)=0,\\ {\overset{⌢}{U}}_{w}\left(rn_{w_{bl}},cn_{w_{bl}}^{\left(j2\right)}\right)=U_{w}\left(rn_{w_{bl}},cn_{w_{bl}}^{\left(j2\right)}\right),\\ {\overset{⌢}{U}}_{\ell }\left(RN_{\ell_{\bar{w}}^{\left(i\right)}},:\right)=r_{\max t}. \end{array}\}\begin{array}{cc} \mathrm{if} & \left|CN_{w_{l}}^{}\right|=0\&j2=1. \end{array}\\ \begin{array}{l} {\overset{⌢}{U}}_{w}\left(\mathrm{end}+1,:\right)=0,\\ {\overset{⌢}{U}}_{w}\left(\mathrm{end},cn_{w_{bl}}^{\left(j2\right)}\right)=U_{w}\left(rn_{w_{bl}},cn_{w_{bl}}^{\left(j2\right)}\right),\\ {\overset{⌢}{U}}_{\ell }\left(\mathrm{end}+1,:\right)=r_{\max t},\\ {\overset{⌢}{U}}_{m}\left(\mathrm{end}+1,:\right)=U_{m}\left(rn_{w_{bl}},:\right),\\ {\overset{⌢}{U}}_{P}\left(\mathrm{end}+1,:\right)=U_{w}\left(rn_{w_{bl}},:\right). \end{array}\}\begin{array}{cc} \mathrm{if} & \left(\left|CN_{w_{l}}^{}\right|=0\&j2>1\right)|\left(\left|CN_{w_{l}}^{}\right|\geq 1\right). \end{array} \end{array}$$

where $\overset{⌢}{U}\left(\mathrm{end},j\right)$ and $\overset{⌢}{U}\left(\mathrm{end}+1,:\right)$ are the same as those in (22).

Note that no state estimate is extracted here, since state estimates are only extracted from newborn components with large weight or from confirmed components. Storing the parameter set in U makes it easy to determine the observation associated with one updated component with the maximum weight, such as $CN_{w_{l}}$ and $CN_{w_{bl}}^{}$.

Case B: $V_{un}\leq \ell_{\bar{w}}^{\left(i\right)}<V_{new}$, the label of unconfirmed components.

① If $\bar{w}\geq w_{l}$ or $\left(w_{m}\leq \bar{w}<w_{l}\right)\&\left(\ell_{\bar{w}}^{\left(i\right)}\in {ℒ}_{k-1,un}\right)\&\left(\ell_{\bar{w}}^{\left(i\right)}\in {ℒ}_{k-2,un}\right)$, new target status information is obtained as follows:

(27) $$\begin{array}{c} r_{\max}=r_{\max}+1,\\ {\hat{X}}_{k}=\left[{\hat{X}}_{k},H_{k}U_{m}(rn^{\left(i\right)},cn^{\left(i\right)})\right],\\ {ℒ}_{k,st}=\left[{ℒ}_{k,st},r_{\max}\right],\\ T_{k,st}=\left[T_{k,st},k\right]. \end{array}$$

where ${ℒ}_{k-1,un}$ is the label set of unconfirmed components with small weights at time k−1, $w_{b}<w_{m}<w_{l}$. In general, $w_{m}$ is set to 0.2.

The information on this new confirmed track is established by modifying ${\overset{⌢}{U}}_{w}$ and ${\overset{⌢}{U}}_{\ell }$ as follows:

(28) $$\begin{array}{c} {\overset{⌢}{U}}_{w}\left(RN_{\ell_{\bar{w}}^{\left(i\right)}},:\right)=0,\\ {\overset{⌢}{U}}_{w}\left(RN_{\ell_{\bar{w}}^{\left(i\right)}},cn^{\left(i\right)}\right)=U_{w}\left(RN_{\ell_{\bar{w}}^{\left(i\right)}},cn^{\left(i\right)}\right),\\ {\overset{⌢}{U}}_{\ell }\left(RN_{\ell_{\bar{w}}^{\left(i\right)}},:\right)=r_{\max}. \end{array}$$

Additionally, $CN=\left[CN,cn^{\left(i\right)}\right]$.

② Otherwise, $\ell_{\bar{w}}^{\left(i\right)}$ is stored in ${ℒ}_{k,un}$, i.e., ${ℒ}_{k,un}=\left[{ℒ}_{k,un},\ell_{\bar{w}}^{\left(i\right)}\right]$.

Case C: $0\leq \ell_{\bar{w}}^{\left(i\right)}<V_{un}$, the label of confirmed components. The single-target state estimate is extracted.

① Preprocessing state estimate. If the observation of one target drifts from its real location, or one target is misdetected at time k−1, the distribution of this target’s posterior information will diverge, and the weights of components associated with its observation at time k will be weakened. Thus, if $\bar{w}<w_{m}$, $rn_{w_{m}}=\arg\underset{i1\in RN_{\ell_{\bar{w}}^{\left(i\right)}}}{\max(}U_{w}\left(i1,1\right))$. Furthermore, if $\left\|H_{k}U_{m}(rn^{\left(i\right)},cn^{\left(i\right)})-H_{k}U_{m}\left(rn_{w_{m}},1\right)\right\|>d\left(1\right)$, $\hat{x}=H_{k}U_{m}\left(rn_{w_{m}},1\right)$. The definition of d(⋅) is provided in Section 3.2.

**Figure A1:**
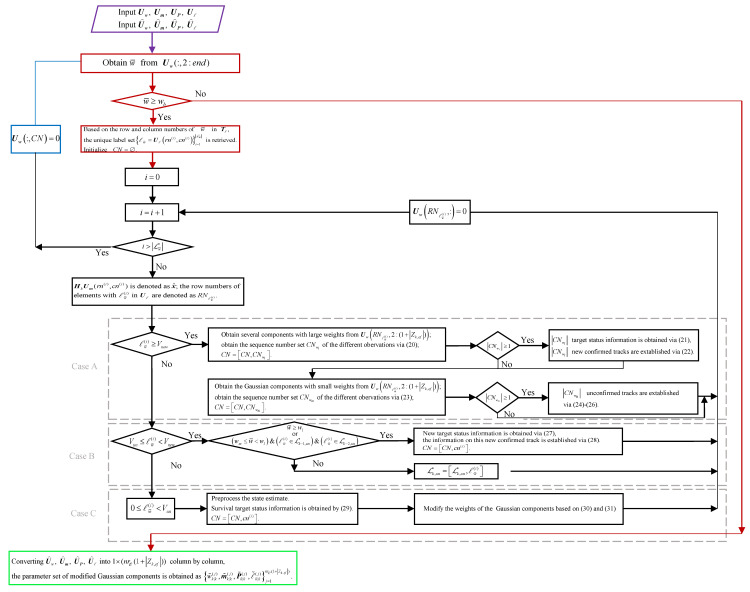
The overall procedure of the state extraction. The operations in the red boxes are step 1, the operations in the black boxes are step 2, the operation in the blue box is step 3, and the operations in the green box are step 4.

**Remark** **1.** *As shown in*Figure A1*, the*1st*column elements in*U*represent the updated information for misdetection at time*k*. When there is some deviation, i.e.,*$\bar{w}<w_{m}$*,*$H_{k}U_{m}\left(rn_{w_{m}},1\right)$*is the optimal state estimate since*$U_{m}\left(rn_{w_{m}},1\right)$*is the update of the posterior component with*$\ell_{\bar{w}}^{\left(i\right)}$*at time*k−1*, and*$U_{w}\left(rn_{w_{m}},1\right)$*is the maximum weight of Gaussian components with*$\ell_{\bar{w}}^{\left(i\right)}$.

② Survival target status information is obtained as follows:

(29) $$\begin{array}{c} {\hat{X}}_{k}=\left[{\hat{X}}_{k},\hat{x}\right],\\ {ℒ}_{k,st}=\left[{ℒ}_{k,st},\ell_{\bar{w}}^{\left(i\right)}\right],\\ T_{k,st}=\left[T_{k,st},k\right]. \end{array}$$

Additionally, $CN=\left[CN,cn^{\left(i\right)}\right]$.

③ Modifying the weights of the Gaussian components. The larger $\bar{w}$ is, the more concentrated the posterior distribution is, and the smaller the radius of the effective region is. The smaller $\bar{w}$ is, the more dispersed the posterior distribution is, and the larger the radius of the effective region is. By calculating the distances between the current $\hat{x}$ and the components with the same label via (30), the weight is modified via (31).

(30) $$d^{\left(i1,j\right)}=\left\|\hat{x}-H_{k}U_{m}\left(RN_{\ell_{\bar{w}}^{\left(i\right)}}^{\left(i1\right)},j\right)\right\|,\forall i1=1,\ldots ,\left|RN_{\ell_{\bar{w}}^{\left(i\right)}}\right|,\forall j=2,\ldots ,1+\left|Z_{k,ef}\right|$$

(31) $$U_{w}\left(RN_{\ell_{\bar{w}}^{\left(i\right)}}^{\left(i1\right)},j\right)=a_{1}\bar{w},\begin{array}{cc} & \left(\bar{w}\geq 0.5\&d^{\left(i1,j\right)}>d\left(3\right)\right)| \end{array}\left(0.4\leq \bar{w}<0.5\&d^{\left(i1,j\right)}>d\left(4\right)\right)|\left(0.2\leq \bar{w}<0.4\&d^{\left(i1,j\right)}>d\left(5\right)\right)|\left(\bar{w}<0.2\&d^{\left(i1,j\right)}>d\left(6\right)\right)$$

In the effective region, the weights of Gaussian components remain unchanged; however, they are reduced to $a_{1}\bar{w}$ outside the region. Here, $a_{1}=0.3$. Dividing the effective region of the posterior distribution based on the weights can further shield against interference, such as detection uncertainty and closely spaced measurements originating from clutter or the other target.

- According to the “one-to-one” principle, the elements in $U\left(RN_{\ell_{\bar{w}}^{\left(i\right)}},:\right)$ are employed in the state extraction; then, they will be discarded as follows:
  (32) $$U_{w}\left(RN_{\ell_{\bar{w}}^{\left(i\right)}},:\right)=0$$

Step 3: Based on the components associated with effective observations, whose sequence numbers are stored in CN, some tracks are established or maintained. Thus, after the $i=1,\ldots ,\left|{ℒ}_{\bar{w}}\right|$ loop is completed, the elements in U(:,CN) will be discarded as follows:

(33) $$U_{w}\left(:,CN\right)=0$$

Next, return to Step 1.

Step 4: Here, $nr_{\overset{⌢}{U}}$ denotes the number of rows in $\overset{⌢}{U}$, ${\overset{⌢}{U}}_{w}$, ${\overset{⌢}{U}}_{m}$, ${\overset{⌢}{U}}_{P}$, and ${\overset{⌢}{U}}_{\ell }$, which all have size $nr_{\overset{⌢}{U}}\times (1+\left|Z_{k,ef}\right|)$. Converting these matrices into $1\times (nr_{\overset{⌢}{U}}(1+\left|Z_{k,ef}\right|))$ column by column, the parameter set of modified Gaussian components is obtained as follows: ${\left\{{\tilde{w}}_{k|k}^{\left(j\right)},{\tilde{m}}_{k|k}^{\left(j\right)},{\tilde{P}}_{k|k}^{\left(j\right)},{\tilde{\ell }}_{k|k}^{\left(j\right)}\right\}}_{j=1}^{nr_{\overset{⌢}{U}}(1+\left|Z_{k,ef}\right|)}$.

This completes the state extraction operation at time k.

The overall procedure of the state extraction of the LGM-PHD filter is given in Appendix A.

**Scheme 1:**
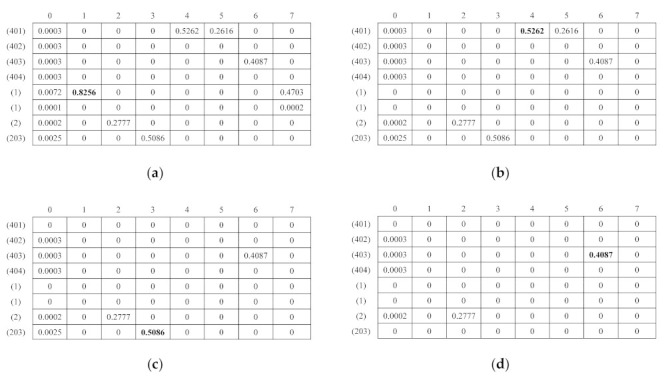

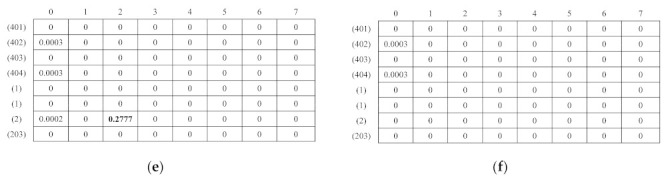
Steps of extracting multiple single-target state estimate based on U. (**a**–**f**): the successive steps of extracting multiple single-target state estimates.

Scheme 1 illustrates the process for extracting multiple single-target state estimates based on U. As the elements in each row in $U_{\ell }$ have the same label, they are recorded at the left of each row in $U_{w}$, as {401}, to simplify the space. In Scheme 1a, at time k, there are two classes of confirmed components: {1,2}; one unconfirmed component: {203}; four classes of newborn components: {401,402,403,404}; seven effective measurements selected via Section 3.2. At time k−1, $r_{\max}$ and $r_{\max t}$ are incremented to $r_{\max}=2$ and $r_{\max t}=203$. Initialize ${\hat{X}}_{k}=\emptyset$, ${ℒ}_{k,st}=\emptyset$, and $T_{k,st}=\emptyset$.

First, the maximum weight in $U_{w}(:,2:end)$ is $\bar{w}=0.8256$, which is $U_{w}\left(5,2\right)$, as shown in Scheme 1a. As $\bar{w}>w_{b}$, obtain $U_{\ell }\left(5,2\right)=1$ and initialize CN=∅. Here, $\left|{ℒ}_{\bar{w}}\right|=1$, $\hat{x}=H_{k}U_{m}\left(5,2\right)$ and $RN_{\ell_{\bar{w}}^{\left(i\right)}}=[5,6]$. As $U_{\ell }\left(5,2\right)<V_{un}$, it is the label of the confirmed component. Then, based on (29), survival target status information is obtained. CN=[CN,2]. Via (32) and (33), $U_{w}\left([5,6],:\right)=0$ and $U_{w}\left(:,2\right)=0$, Scheme 1b is obtained. It is obvious that the interference of $U_{w}\left(5,8\right)$ caused by $z_{k,ef}^{\left(7\right)}$ with state estimation is prevented, which represents the clutter close to $z_{k,ef}^{\left(1\right)}$.

Second, the maximum weight in $U_{w}(:,2:end)$ is $\bar{w}=0.5262$, which is $U_{w}\left(1,5\right)$, as shown in Scheme 1b. As $\bar{w}>w_{b}$, obtain $U_{\ell }\left(1,5\right)=401$ and initialize CN=∅. Here, $\left|{ℒ}_{\bar{w}}\right|=1$, $\hat{x}=H_{k}U_{m}\left(1,5\right)$ and $RN_{\ell_{\bar{w}}^{\left(i\right)}}=1$. As $U_{\ell }\left(1,5\right)>V_{new}$, it is the label of the newborn component. Based on (20), $CN_{w_{l}}=5$, and CN=[CN,5]. Then, information on one new target status is established via (21), and $r_{\max}$ is incremented to 3. The information on this new confirmed track is established by (22).

Additionally, based on (23), $CN_{w_{bl}}=6$, and CN=[CN,6]. Then, one new unconfirmed track is established via (24–26). Here, $r_{\max t}$ is incremented to 204. Via (32) and (33), $U_{w}\left(1,:\right)=0$ and $U_{w}\left(:,[5,6]\right)=0$, Scheme 1c is obtained.

Third, the maximum weight in $U_{w}(:,2:end)$ is $\bar{w}=0.5086$, which is $U_{w}\left(8,4\right)$, as shown in Scheme 1c. As $\bar{w}>w_{b}$, obtain $U_{\ell }\left(8,4\right)=203$ and initialize CN=∅. Here, $\left|{ℒ}_{\bar{w}}\right|=1$, $\hat{x}=H_{k}U_{m}\left(8,4\right)$ and $RN_{\ell_{\bar{w}}^{\left(i\right)}}=8$. As $V_{un}<U_{\ell }\left(8,4\right)\leq V_{new}$, it is the label of the unconfirmed component. Since $\bar{w}>w_{l}$, a new target status information is obtained by (27) and CN=[CN,4]. Via (32) and (33), $U_{w}\left(8,:\right)=0$ and $U_{w}\left(:,4\right)=0$, Scheme 1d is obtained.

Fourth, the maximum weight in $U_{w}(:,2:end)$ is $\bar{w}=0.4087$, which is $U_{w}\left(3,7\right)$, as shown in Scheme 1d. As $\bar{w}>w_{b}$, obtain $U_{\ell }\left(3,7\right)=403$ and initialize CN=∅. Here, $\left|{ℒ}_{\bar{w}}\right|=1$, $\hat{x}=H_{k}U_{m}\left(3,7\right)$ and $RN_{\ell_{\bar{w}}^{\left(i\right)}}=3$. As $U_{\ell }\left(3,7\right)>V_{new}$, it is the label of the newborn component. Based on (20), $CN_{w_{l}}=7$, and CN=[CN,7]. Then, information on one new target status is established via (21), and $r_{\max}$ is incremented to 4. The information on this new confirmed track is established by (22). Via (32) and (33), $U_{w}\left(3,:\right)=0$ and $U_{w}\left(:,7\right)=0$, Scheme 1e is obtained.

Fifth, the maximum weight in $U_{w}(:,2:end)$ is $\bar{w}=0.2777$, which is $U_{w}\left(7,3\right)$, as shown in Scheme 1e. As $\bar{w}>w_{b}$, obtain $U_{\ell }\left(7,3\right)=2$ and initialize CN=∅. Here, $\left|{ℒ}_{\bar{w}}\right|=1$, $\hat{x}=H_{k}U_{m}\left(7,3\right)$ and $RN_{\ell_{\bar{w}}^{\left(i\right)}}=7$. As $U_{\ell }\left(7,3\right)<V_{un}$, it is the label of the confirmed component. Then, based on (29), survival target status information is obtained. CN=[CN,3]. Via (32) and (33), $U_{w}\left(7,:\right)=0$ and $U_{w}\left(:,3\right)=0$, Scheme 1f is obtained.

Sixth, the maximum weight in $U_{w}(:,2:end)$ is $\bar{w}=0.0003$ in Scheme 1f. As $\bar{w}<w_{b}$, step 1 to step 3 of the state extraction at time k is completed. ${\hat{X}}_{k}$, ${ℒ}_{k,st}$, and $T_{k,st}$ are all updated.

**Remark** **2.** *In the proposed filter, since the components approximating one survival target can be identified, the weight threshold for state extraction is reduced compared with the threshold in the basic GM-PHD filter. This ensures that the survival target can be tracked when the proper measurement error occurs, such as* $U_{w}\left(7,3\right)=0.2777$ *in Scheme 1. Additionally, only one state estimate can be extracted from the survival Gaussian components with the same label. This ensures that the interference of clutter on the number of state estimates, such as* $U_{w}\left(5,8\right)=0.4703$ *in Scheme 1, can be prevented.*

**Remark** **3.**  *The behavior of labeling state estimates in the proposed filter is determined by employing the weights and labels of the updated components, which requires no additional computational processing. Furthermore, the updating process and query tables in the state extraction algorithm are all based on the selected observations. Thus, the real-time performance of the LGM-PHD filter is guaranteed.*

### 3.5. Pruning

To reduce the computational cost, reducing the number of Gaussian components by pruning is necessary.

Step 1: Prune confirmed and unconfirmed components that have no state estimates for successive $n_{no}$ time steps. In our filter, $n_{no}=6$.

① The label set ${ℒ}_{k,no}$ of confirmed and unconfirmed components with no state estimates at time k is:

(34) $${ℒ}_{k,no}={\left\{\ell_{k,no}^{\left(i\right)}\right\}}_{i=1}^{\left|{ℒ}_{k,no}\right|}=uni({\left\{{\tilde{\ell }}_{k|k}^{\left(j\right)}\right\}}_{j=1}^{nr_{\overset{⌢}{U}}(1+\left|Z_{k,ef}\right|)})-{\left\{\ell_{\gamma ,k}^{\left(i\right)}\right\}}_{i=1}^{J_{\gamma ,k}}-{ℒ}_{k,st}$$

② For $i=1,\ldots ,\left|{ℒ}_{k,no}\right|$, check the occurrence number of $\ell_{k,no}^{\left(i\right)}$ in ${\left\{{ℒ}_{k1,no}\right\}}_{k1=k-n_{no}+1}^{k-1}$. If $\ell_{k,no}^{\left(i\right)}$ appears in ${ℒ}_{k1,no}$ at each time $k1=k-n_{no}+1,\ldots ,k-1$, ${\left\{{\tilde{w}}_{k|k}^{\left(j\right)}=0|{\tilde{\ell }}_{k|k}^{\left(j\right)}=\ell_{k,no}^{\left(i\right)}\right\}}_{j=1}^{nr_{\overset{⌢}{U}}(1+\left|Z_{k,ef}\right|)}$. This procedure improves accuracy.

Step 2: Based on the truncation threshold $w_{et}=10^{-5}$, a certain number of the components with the strongest weights are retained in I,

(35) $$I=\left\{j=1,\ldots ,nr_{\overset{⌢}{U}}(1+\left|Z_{k,ef}\right|)|{\tilde{w}}_{k|k}^{\left(j\right)}>w_{et}\right\}$$

Step 3: Obtain ${\left\{{\overset{⌢}{w}}_{k|k}^{\left(j\right)},{\overset{⌢}{m}}_{k|k}^{\left(j\right)},{\overset{⌢}{P}}_{k|k}^{\left(j\right)},{\overset{⌢}{\ell }}_{k|k}^{\left(j\right)}\right\}}_{j=1}^{J_{k|k}}$, where $J_{k|k}=\left|I\right|$, $\left({\overset{⌢}{w}}_{k|k}^{\left(j\right)},{\overset{⌢}{m}}_{k|k}^{\left(j\right)},{\overset{⌢}{P}}_{k|k}^{\left(j\right)},{\overset{⌢}{\ell }}_{k|k}^{\left(j\right)}|j=1)=({\tilde{w}}_{k|k}^{\left(j\right)},{\tilde{m}}_{k|k}^{\left(j\right)},{\tilde{P}}_{k|k}^{\left(j\right)},{\tilde{\ell }}_{k|k}^{\left(j\right)}|j=I(1)\right)$, among others.

### 3.6. Merging and Capping

In the basic GM-PHD filter, some of the Gaussian components that are very close together will be approximated by a single Gaussian [32]. This means that, in the merging procedure, the posterior information of targets in close proximity will interfere. This will result in the loss of posterior information, approximated by components with small weights, of some targets. To avoid such loss, in the proposed filter, only the components with the same label can be merged.

First, let $d_{Th}$ be the merging threshold. In general, $d_{Th}=4$. Set ${J^{\prime }}_{k}=0$, and $I1={\left\{i1\right\}}_{i1=1}^{J_{k|k}}$. The specific steps in merging process are as follows.

Step 1: Let ${J^{\prime }}_{k}={J^{\prime }}_{k}+1$, $j=\arg\underset{i1\in I1}{\max}\left({\overset{⌢}{w}}_{k|k}^{\left(i1\right)}\right)$. Then, ${\overset{⌢}{\ell }}_{k|k}^{\left(j\right)}$ is obtained.

Step 2: The sequence number set I2 is given as follows:

(36) $$I2=\{i1\in I1|{\overset{⌢}{\ell }}_{k|k}^{\left(i1\right)}={\overset{⌢}{\ell }}_{k|k}^{\left(j\right)},{\left({\overset{⌢}{m}}_{k|k}^{\left(i1\right)}-{\overset{⌢}{m}}_{k|k}^{\left(j\right)}\right)}^{T}{\left({\overset{⌢}{P}}_{k|k}^{\left(i1\right)}\right)}^{-1}({\overset{⌢}{m}}_{k|k}^{\left(i1\right)}-{\overset{⌢}{m}}_{k|k}^{\left(j\right)})\leq d_{Th}\}$$

Step 3: Then, a merged Gaussian component is given as follows:

(37) $${\overset{⌣}{w}}_{k}^{\left({J^{\prime }}_{k}\right)}=\sum_{i1\in I2}{\overset{⌢}{w}}_{k|k}^{\left(i1\right)}$$

(38) $${\overset{⌣}{m}}_{k}^{\left({J^{\prime }}_{k}\right)}=\frac{1}{{\overset{⌣}{w}}_{k}^{\left(J_{k}\right)}}\sum_{i1\in I2}{\overset{⌢}{w}}_{k|k}^{\left(i1\right)}{\overset{⌢}{m}}_{k|k}^{\left(i1\right)}$$

(39) $${\overset{⌣}{P}}_{k}^{\left({J^{\prime }}_{k}\right)}=\frac{1}{{\overset{⌣}{w}}_{k}^{\left({J^{\prime }}_{k}\right)}}\sum_{i1\in I2}{\overset{⌢}{w}}_{k|k}^{\left(i1\right)}\left({\overset{⌢}{P}}_{k|k}^{\left(i1\right)}+\left({\overset{⌣}{m}}_{k}^{\left({J^{\prime }}_{k}\right)}-{\overset{⌢}{m}}_{k|k}^{\left(i1\right)}\right){\left({\overset{⌣}{m}}_{k}^{\left({J^{\prime }}_{k}\right)}-{\overset{⌢}{m}}_{k|k}^{\left(i1\right)}\right)}^{T}\right)$$

(40) $${\overset{⌣}{\ell }}_{k}^{\left({J^{\prime }}_{k}\right)}={\overset{⌢}{\ell }}_{k|k}^{\left(j\right)}$$

Step 4: Execute I1=I1−I2. If I1 is not empty, skip to Step 1.

Step 5: The output of merging procedure is obtained as follows: ${\left\{{\overset{⌣}{w}}_{k}^{\left(j\right)},{\overset{⌣}{m}}_{k}^{\left(j\right)},{\overset{⌣}{P}}_{k}^{\left(j\right)},{\overset{⌣}{\ell }}_{k}^{\left(j\right)}\right\}}_{j=1}^{{J^{\prime }}_{k}}$.

Next, if ${J^{\prime }}_{k}>J_{\max1}$, replace ${\left\{{\overset{⌣}{w}}_{k}^{\left(j\right)},{\overset{⌣}{m}}_{k}^{\left(j\right)},{\overset{⌣}{P}}_{k}^{\left(j\right)},{\overset{⌣}{\ell }}_{k}^{\left(j\right)}\right\}}_{j=1}^{{J^{\prime }}_{k}}$ by those of the $J_{\max1}$ Gaussian components with the largest weights, and the posterior intensity for propagating is obtained as follows: ${\left\{w_{k}^{\left(j\right)},m_{k}^{\left(j\right)},P_{k}^{\left(j\right)},\ell_{k}^{\left(j\right)}\right\}}_{j=1}^{J_{k}}$. In this paper, $J_{\max1}=150$.

### 3.7. Steps in the Proposed Algorithm

For completeness, we summarized the key steps of the LGM-PHD filter as follows.

Step 1: Prediction. The predicted components ${\left\{w_{k|k-1}^{\left(i\right)},m_{k|k-1}^{\left(i\right)},P_{k|k-1}^{\left(i\right)}\right\}}_{i=1}^{J_{k|k-1}}$ are obtained via (4) and their labels are obtained via (16). Then, obtain the parameter set ${\left\{w_{k|k-1}^{\left(i\right)},m_{k|k-1}^{\left(i\right)},P_{k|k-1}^{\left(i\right)},\ell_{k|k-1}^{\left(i\right)}\right\}}_{i=1}^{J_{k|k-1}}$ of the predicted components.

Step 2: Observation selection. The effective observation set $Z_{k,ef}$ is obtained by eliminating the clutter in the low prior region via Algorithm 1.

Step 3: Updating. Based on $Z_{k,ef}$, the predicted components are updated via (5) to (12), and the parameters w, m, P, and ℓ associated with each component are stored in U and $\overset{⌢}{U}$, respectively.

Step 4: State extraction. As per the steps in 3.4, based on U and $\overset{⌢}{U}$, the multitarget state extraction is converted into multiple single-target state extractions that can provide the identity label, and the occurrence time for each estimate, i.e., ${\hat{X}}_{k}$, ${ℒ}_{k,st}$, and $T_{k,st}$ are obtained. Then, converting the four $\overset{⌢}{U}$ values into $1\times (nr_{\overset{⌢}{U}}(1+\left|Z_{k,ef}\right|))$ column by column, the parameter set ${\left\{{\tilde{w}}_{k|k}^{\left(j\right)},{\tilde{m}}_{k|k}^{\left(j\right)},{\tilde{P}}_{k|k}^{\left(j\right)},{\tilde{\ell }}_{k|k}^{\left(j\right)}\right\}}_{j=1}^{nr_{\overset{⌢}{U}}(1+\left|Z_{k,ef}\right|)}$ of the modified Gaussian components is obtained.

Step 5: Pruning. As per the steps in Section 3.5, ${\left\{{\overset{⌢}{w}}_{k|k}^{\left(j\right)},{\overset{⌢}{m}}_{k|k}^{\left(j\right)},{\overset{⌢}{P}}_{k|k}^{\left(j\right)},{\overset{⌢}{\ell }}_{k|k}^{\left(j\right)}\right\}}_{j=1}^{J_{k|k}}$ are obtained.

Step 6: Merging and capping. The closely spaced components with the same label are merged as per the steps in Section 3.6 to obtain ${\left\{w_{k}^{\left(j\right)},m_{k}^{\left(j\right)},P_{k}^{\left(j\right)},\ell_{k}^{\left(j\right)}\right\}}_{j=1}^{J_{k}}$ for propagation.

After Step 4, based on ${\hat{X}}_{k}$, ${ℒ}_{k,st}$, and $T_{k,st}$, explicit track maintenance is achieved by connecting the state estimates with the same label in sequence.

**Remark** **4.**  *The proposed filter does not specifically define track disappearance, and each track is left as it is, whether it has disappeared or been misdetected, except that no state estimate appears in seven successive time steps. When a target has been redetected, as long as confirmed Gaussian components for this target are still available for propagation, this target can still have clues to be tracked, and the state estimate will be extracted from these components only if its posterior information satisfies the necessary conditions.*

## 4. Simulation and Results Analysis

The experiment presented in this section aimed to test the performance of the proposed filter. We compared the proposed filter with the basic GM-PHD filter [32] and the Gibbs-GLMB filter [10]. Without loss of generality, we employed the MTT simulation model given in [39]. In a two-dimensional scenario over the region $\left[\begin{array}{cc} -1000 & 1000 \end{array}\right]\;m\times \left[\begin{array}{cc} -1000 & 1000 \end{array}\right]\;m$, each target moved according to the following linear Gaussian dynamics:

$$x_{k}=\left[\begin{array}{l} \begin{array}{cccc} 1 & \Delta & 0 & 0 \end{array}\\ \begin{array}{cccc} 0 & 1 & 0 & 0 \end{array}\\ \begin{array}{cccc} 0 & 0 & 1 & \Delta \end{array}\\ \begin{array}{cccc} 0 & 0 & 0 & 1 \end{array} \end{array}\right]x_{k-1}+\sigma_{\omega }^{2}\left[\begin{array}{cccc} \Delta^{2}/4 & \Delta^{3}/2 & 0 & 0\\ \Delta^{3}/2 & \Delta^{2} & 0 & 0\\ 0 & 0 & \Delta^{2}/4 & \Delta^{3}/2\\ 0 & 0 & \Delta^{3}/2 & \Delta^{2} \end{array}\right]$$

where $x_{k}={\left[\begin{array}{cccc} x_{1,k} & x_{2,k} & x_{3,k} & x_{4,k} \end{array}\right]}^{T}$, ${\left[\begin{array}{cc} x_{1,k} & x_{3,k} \end{array}\right]}^{T}$, and ${\left[\begin{array}{cc} x_{2,k} & x_{4,k} \end{array}\right]}^{T}$ are, respectively, the position and velocity of the target at time k. Δ=1 s  is the sampling time, and $\sigma_{\omega }=5{\;m/s}^{2}$. Targets can appear and disappear in the scene at any time with the survival probability $p_{S,k}=0.95$. For the Gibbs-GLMB filter, the birth model is a labeled multi-Bernoulli with parameters ${\left\{r_{B,k}\left(\ell_{i}\right),p_{B,k}\left(\ell_{i}\right)\right\}}_{i=1}^{4}$, where $\ell_{i}=(k,i)$, $r_{B,k}\left(\ell_{i}\right)=0.03$ and $p_{B,k}\left(\ell_{i}\right)=N\left(x;m_{B}^{\left(i\right)},P_{B}\right)$ with $m_{B}^{\left(1\right)}={\left[\begin{array}{cccc} 0 & 0 & 0 & 0 \end{array}\right]}^{T}$, $m_{B}^{\left(2\right)}={\left[\begin{array}{cccc} 400 & 0 & -600 & 0 \end{array}\right]}^{T}$, $m_{B}^{\left(3\right)}={\left[\begin{array}{cccc} -800 & 0 & -200 & 0 \end{array}\right]}^{T}$, $m_{B}^{\left(4\right)}={\left[\begin{array}{cccc} -200 & 0 & 800 & 0 \end{array}\right]}^{T}$, and $P_{B}=diag{\left({\left[\begin{array}{cccc} 10 & 10 & 10 & 10 \end{array}\right]}^{T}\right)}^{2}$. For the GM-PHD filter, the newborn targets appear spontaneously according to a Poisson point process with intensity function $\gamma_{k}\left(x_{k}\right)=\sum_{i=1}^{4}w_{\gamma ,k}^{\left(i\right)}N\left(x;m_{\gamma ,k}^{\left(i\right)},P_{\gamma ,k}^{\left(i\right)}\right)$, which are given by $m_{\gamma ,k}^{\left(1\right)}=m_{B}^{\left(1\right)}$, $m_{\gamma ,k}^{\left(2\right)}=m_{B}^{\left(2\right)}$, $m_{\gamma ,k}^{\left(3\right)}=m_{B}^{\left(3\right)}$, $m_{\gamma ,k}^{\left(4\right)}=m_{B}^{\left(4\right)}$, $P_{\gamma ,k}=P_{B}$, and $w_{\gamma ,k}^{\left(i\right)}=r_{B,k}\left(\ell_{i}\right)$. Additionally, $\ell_{\gamma ,k}^{\left(1\right)}=401$, $\ell_{\gamma ,k}^{\left(2\right)}=402$, $\ell_{\gamma ,k}^{\left(3\right)}=403$, and $\ell_{\gamma ,k}^{\left(4\right)}=404$.

The linear target-originated measurement is given as follows:

$$y_{k}=\left[\begin{array}{l} \begin{array}{cccc} 1 & 0 & 0 & 0 \end{array}\\ \begin{array}{cccc} 0 & 0 & 1 & 0 \end{array} \end{array}\right]x_{k}+\left[\begin{array}{l} \upsilon_{x,k}\\ \upsilon_{y,k} \end{array}\right]$$

where $\upsilon_{x,k}$ and $\upsilon_{y,k}$ represent mutually independent zero-mean Gaussian white noise with standard deviations of $\sigma_{x}=10\;m\;$ and $\sigma_{y}=10\;m\;$, respectively. The clutter is uniformly distributed over the region $\left[\begin{array}{cc} -1000 & 1000 \end{array}\right]\;m\times \left[\begin{array}{cc} -1000 & 1000 \end{array}\right]\;m$ with an average rate of λ points per scan, i.e., $\kappa =\lambda /2000^{2}$, for the basic GM-PHD filter. To ensure a fair comparison, the basic GM-PHD filter employs the gating algorithm that is used in Gibbs-GLMB filter, and $G_{th}=9$.

The maximum number of Gaussian components is $J_{\max}=100$ in the proposed filter and the basic GM-PHD filter. The optimal subpattern assignment (OSPA) metric [40] is used to evaluate the tracking performance of different filters, and the order and cut-off are set as p=1 and c=100, respectively. The presented result is the average of 250 independent repeated experiments but with independently generated observations for each trial. All experiments were run in MATLAB R2013b on a computer with a 3.3 GHz CPU and 8 GB RAM.

**Figure 4:**
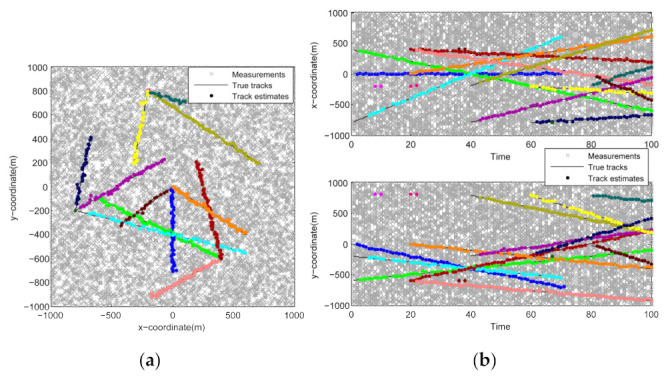
Track estimates of the proposed filter and ground truths for a sample run ($\lambda =100,p_{D,k}=0.9$). (**a**) The true and estimated trajectories of targets. Different trajectories estimated by the proposed filter are denoted by different colored dots. (**b**) The true and estimated trajectories of targets in x and y coordinates, respectively. © 2021 IEEE.

**Figure 5:**
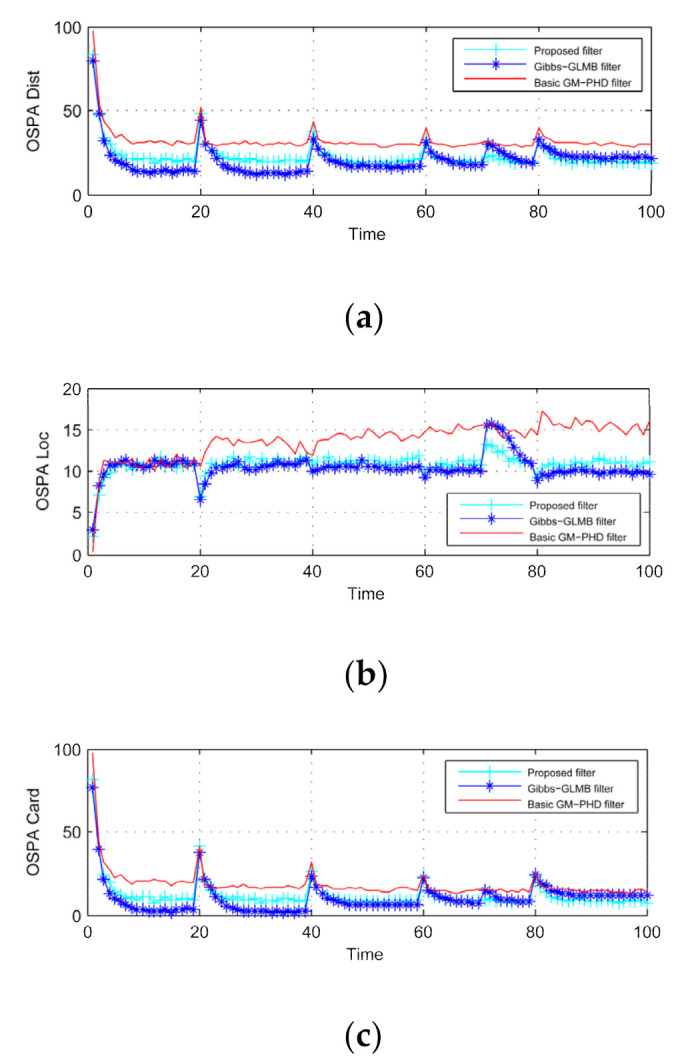
Comparison of different algorithms for 250 sample runs ($\lambda =100,p_{D,k}=0.9$). (**a**) Average Optimal subpattern assignment (OSPA) distances of different filters; (**b**) OSPA locations of different filters; (**c**) OSPA cardinalities of different filters.

**Figure 6:**
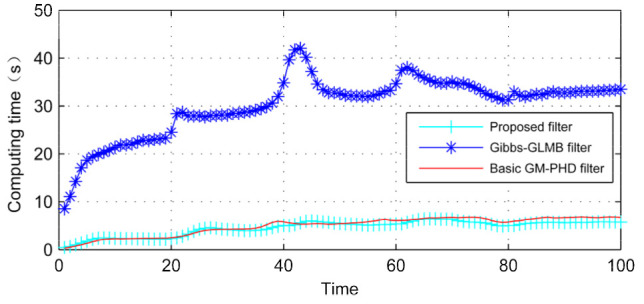
Computing times of different algorithms for 250 sample runs ($\lambda =100,p_{D,k}=0.9$).

Example 1: Detailed performance comparison under λ=100 and $p_{D,k}=0.9$. As shown in Figure 4, Figure 5 and Figure 6, the number of clutter points is λ=100, and the detection probability is $p_{D,k}=0.9$. Figure 4 shows the track estimates obtained from the proposed filter for a single trial [38]. The average OSPA errors and computing time for 250 sample runs of each filter are shown in Figure 5 and Figure 6.

Figure 4a shows that the proposed filter can achieve correct trajectory maintenance in MTT in the presence of dense clutter. As the proposed filter does not supplement the state estimate for the misdetected target, there are discontinuities in the tracks. However, this does not affect the trajectory continuity, and the redetected targets can be tracked in time. Additionally, as shown in Figure 4b, one false track is around $H_{k}m_{\gamma ,k}^{\left(2\right)}$, one false track is around $H_{k}m_{\gamma ,k}^{\left(3\right)}$ and two false tracks are around $H_{k}m_{\gamma ,k}^{\left(4\right)}$. This is because the proposed filter has no special procedure to account for the false estimates around the newborn locations. Similarly, they do not affect the continuity of other established tracks, and will disappear over time.

In Figure 5, the average OSPA distances of the proposed filter, the Gibbs-GLMB filter and the basic GM-PHD filter were 22.522 m, 20.497 m, and 31.990 m, respectively. Compared with the basic GM-PHD filter, the average tracking performance of the proposed filter and the Gibbs-GLMB filter increased by 29.60% and 35.92%, respectively. Clearly, the tracking performance of the LGM-PHD filter was slightly lower than that of the Gibbs-GLMB filter. This is mainly caused by the phenomena shown in Figure 4b. However, this did not affect the survival target tracking.

As shown in Figure 6, the average computing times in seconds of a single run (100 time steps) of our MATLAB implementations were 4.60 s (the proposed filter), 30.21 s (the Gibbs-GLMB filter), and 4.96 s (the basic GM-PHD filter), respectively. Compared with the basic GM-PHD filter, the real-time performance of the proposed filter and the Gibbs-GLMB filter increased by 7.26% and −509%, respectively. As expected, the real-time performance of the proposed filter was better than that of the Gibbs-GLMB filter.

**Figure 7:**
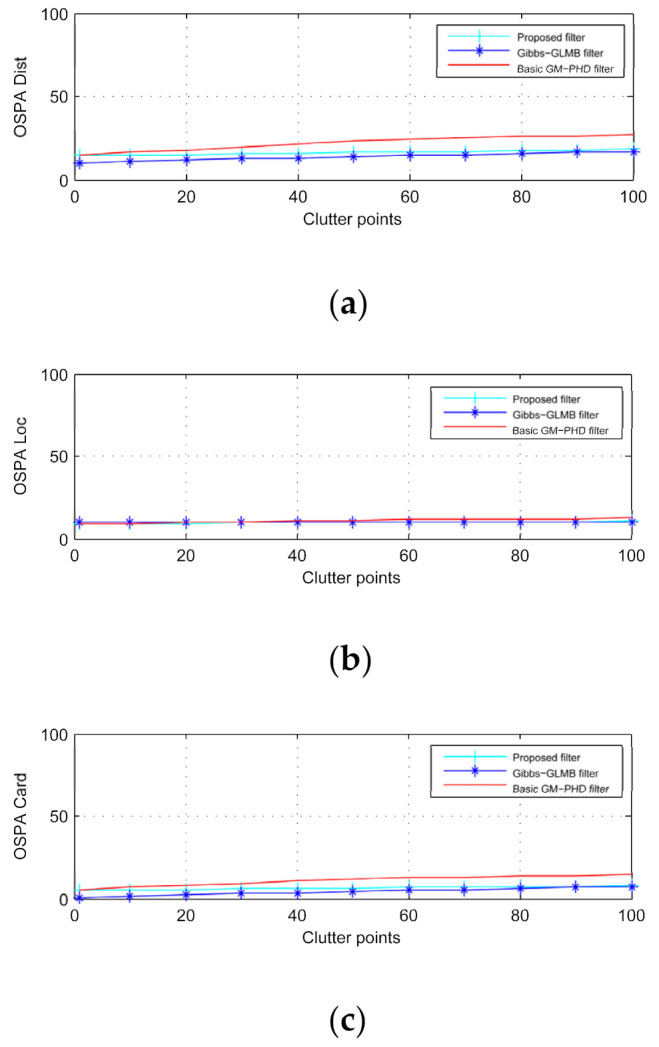
Comparison of different algorithms under various clutter rates for 250 sample runs ($p_{D,k}=0.95$). (**a**) Average OSPA distances of different filters; (**b**) OSPA locations of different filters; (**c**) OSPA cardinalities of different filters.

**Figure 8:**
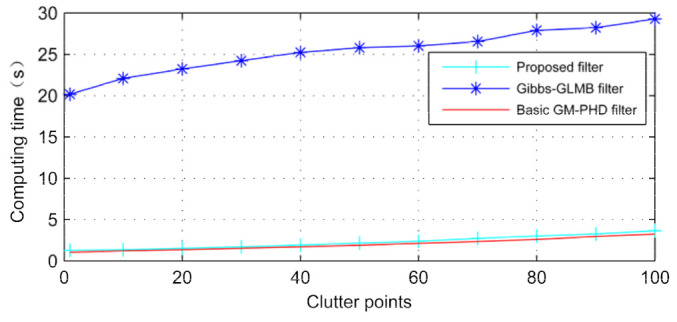
Computing times of different algorithms under various clutter rates for 250 sample runs ($p_{D,k}=0.95$).

Example 2: Performance comparison under various clutter rates for $p_{D,k}=0.95$. In this example, the performance of the proposed filter was evaluated with various clutter rates. The clutter rate λ changed from 1 to 100, and the probability of detection was set to $p_{D,k}=0.95$. Figure 7 shows the comparison results in terms of the average OSPA errors for 250 sample runs of the different algorithms under different clutter rates, and their computing times are shown in Figure 8.

From Figure 7 and Figure 8, we can see that the proposed filter achieved a better OSPA distance than the basic GM-PHD filter and kept a similar real-time performance as the clutter rate increased. Compared with the basic GM-PHD filter, with λ=20, the average tracking performances of the proposed filter and the Gibbs-GLMB filter increased by 15.75% and 33.14%, respectively; with λ=100, they increased by 31.96% and 37.14%, respectively. The advantage of the proposed filter can be fully demonstrated in the dense clutter environment.

**Figure 9:**
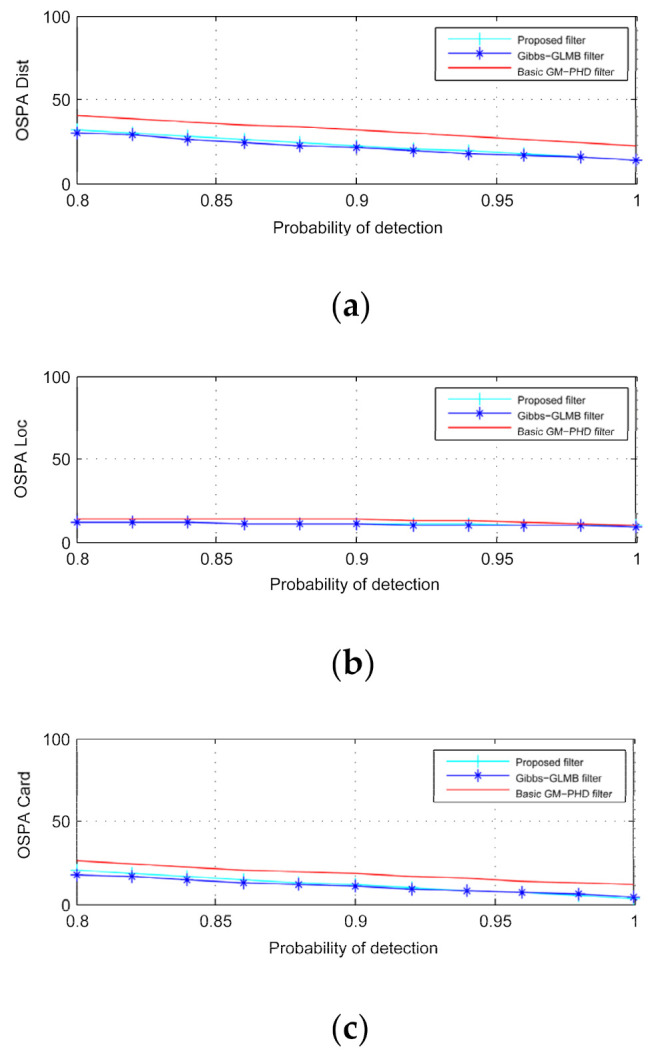
Comparison of different algorithms under various probabilities of detection for 250 sample runs (λ=100). (**a**) Average OSPA distances of different filters; (**b**) OSPA locations of different filters; (**c**) OSPA cardinalities of different filters.

**Figure 10:**
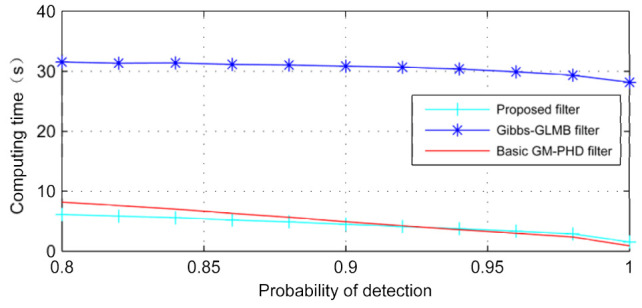
Computing times of different algorithms under various probabilities of detection for 250 sample runs (λ=100 ).

Example 3: Performance comparison under various probabilities of detection for λ=100. In this example, various probabilities of detection were employed to evaluate the effectiveness of the proposed filter. The probability of detection $p_{D,k}$ changed from 0.8 to 1, and the clutter rate was kept unchanged at λ=100. The comparison results of the average OSPA errors for 250 sample runs of the different algorithms under different probabilities of detection are shown in Figure 9. Figure 10 shows the corresponding computing times of each filter.

Clearly, the proposed filter achieved a better OSPA distance than the basic GM-PHD filter and maintained a similar real-time performance at each probability of detection. As the probability of detection increased, the OSPA distance of the proposed filter decreased. Compared with the basic GM-PHD filter, with $p_{D,k}=0.84$, the average tracking performances of the proposed filter and the Gibbs-GLMB filter increased by 23.03% and 29.13%, respectively; with $p_{D,k}=0.96$, they increased by 33.21% and 35.79%, respectively.

## 5. Conclusions

The paper proposed a computationally efficient LGM-PHD filter that can explicitly accommodate the estimation of target trajectories in MTT. Based on the proposed query matrices, state estimates with identity labels and occurrence times were extracted, and no additional associated procedures were required. Three numerical and simulations analyses demonstrated that the LGM-PHD filter could effectively improve the tracking performance as compared with the basic GM-PHD filter, which was slightly lower than that of the Gibbs-GLMB filter. As the proposed filter could maintain a similar to or slightly lower real-time performance than that of the basic GM-PHD filter, it could be an alternative filter explicitly accommodating target trajectories in real-time processing applications. We should point out that the LGM-PHD filter is not suitable for low detection scenarios. In our future work, we will investigate multisensor technology [12,13,39] for improving tracking performance.

## Data Availability

Not applicable.
